# Presence of Multi-Morbidities and Colorectal Cancer Screening Utilization among Breast Cancer Survivors

**DOI:** 10.3390/cancers15072077

**Published:** 2023-03-30

**Authors:** Meng-Han Tsai, Caitlyn Grunert, Jacqueline B. Vo, Justin X. Moore, Avirup Guha

**Affiliations:** 1 Cancer Prevention, Control & Population Health Program, Georgia Cancer Center, Department of Medicine, Medical College of Georgia, Augusta University, Augusta, GA 30912, USA; 2 Georgia Prevention Institute, Augusta University, Augusta, GA 30912, USA; 3 Division of Cancer Epidemiology & Genetics, National Cancer Institute, Bethesda, MD 20814, USA; 4 Institute of Public and Preventive Health, Augusta University, Augusta, GA 30912, USA; 5 Cardio-Oncology Program, Georgia Cancer Center, Division of Cardiology, Department of Medicine, Cardiology, Medical College of Georgia, Augusta University, Augusta, GA 30912, USA

**Keywords:** colorectal cancer screening, breast cancer survivors, chronic disease conditions, quality of life

## Abstract

**Simple Summary:**

Breast cancer survivors have an increased risk of developing colorectal cancer (CRC) due to shared risk factors; thus, adherence to CRC screening recommendation should be prioritized for breast cancer survivors. Further, breast cancer survivors with multiple chronic diseases commonly experience poorer survival outcomes. These together highlight the need for evaluation and management of their chronic disease conditions. Understanding their chronic disease conditions may assist providers offering appropriate screening recommendations for CRC. Through our cross-sectional study, we sought to examine the association between the presence of multiple chronic disease conditions (e.g., diabetes and coronary heart disease) and guideline-concordant CRC screening utilization. Further, we determined the factors in sociodemographic characteristics, quality of life, and cancer-related factors associated with screening uptake among breast cancer survivors. Findings from this study suggest the importance of chronic disease management considering mental/physical health status and other factors (race/ethnicity or receipt of follow-up care plans) in cancer survivorship care.

**Abstract:**

Purpose: Our study aimed to examine the association between the presence of chronic diseases with guideline-concordant colorectal cancer (CRC) screening utilization among breast cancer survivors. Methods: We analyzed data among women with a history of breast cancer from the 2016, 2018, and 2020 Behavioral Risk Factor Surveillance System. Receipt of guideline-concordant CRC screening was the outcome of interest. Diabetes, coronary heart disease/myocardial infarction, stroke, chronic obstructive pulmonary disease, emphysema/chronic bronchitis, arthritis, depressive disorder, or kidney diseases were included in chronic disease conditions. Results: Among 1324 survivors, those with multi-morbidities (3+ chronic diseases; 88.3%) had higher CRC screening use compared to those with one (84.4%) or two (85.4%) diseases (*p*-value < 0.05). In multivariable analysis, survivors with multi-morbidities were two times more likely to have CRC screening compared to those with only one disease (OR, 2.10; 95% CI, 1.11–3.98). Among survivors with multi-morbidities, Black women (OR, 14.07; 95% CI, 5.61–35.27), and those with frequent poor physical health (OR, 3.32; 95% CI, 1.57–7.00) were positively associated with CRC screening use. Conversely, survivors with frequent poor mental health were 67% less likely to receive CRC screening (OR, 0.33; 95% CI, 0.14–0.74). Conclusion: Among breast cancer survivors, multi-morbidities were positively associated with CRC screening.

## 1. Introduction

In 2022, more than 4 million women living in the United States (US) had a history of invasive breast cancer [1,2]. Breast cancer survivors have an increased risk of obtaining other illnesses compared to women without a history of cancer. Evidence shown that breast cancer survivors are especially vulnerable to secondary cancers [3], with 2.5-fold increased odds of developing colorectal cancer (CRC) compared to those without a history of cancer [3]. This may be attributed to the carcinogenetic effects of primary-cancer related treatment (e.g., chemotherapy or radiation) [4], genetic predisposition [5], and personal behaviors [6]. However, the development of secondary CRC after breast cancer can be a life-threatening event for breast cancer survivors [7]. Thus, adherence to CRC screening recommendations should be prioritized for breast cancer survivors.

Further, breast cancer survivors commonly suffer from several chronic disease conditions (e.g., type 2 diabetes and hypertension) [8] due to shared risk factors such as smoking and obesity [9]. Multi-morbidities refer to the presence of multiple chronic medical conditions, typically defined as two or more concurrent chronic diseases or conditions in an individual. Poorer survival outcomes among breast cancer patients with multi-morbidities have been established [10] despite improved five-year cancer survival rates in the US [1]. Thus, the presence of multi-morbidities has been linked to increased healthcare utilization in breast cancer survivors [11], including CRC screening uptake for secondary cancer prevention [12]. Such evidence highlights the need for evaluation and management of breast cancer survivors’ chronic disease conditions. Understanding their chronic disease conditions may assist providers offering appropriate screening recommendations for CRC.

In addition to chronic disease conditions, other factors such as sociodemographic characteristics (i.e., race/ethnicity) and receipt of survivorship care plans are related to CRC screening use among cancer survivors [13,14]. A prior study observed that Hispanic survivors were less likely to be screened for CRC [13]. Factors associated with low screening use include worse quality of life and low income among Hispanic survivors [15]. Conversely, greater CRC screening use was found when survivors received a survivorship care plan in breast, cervical, colorectal, prostate, melanoma, and other cancers [13]. Recent efforts were made toward survivors’ quality of life, including integrating information on multi-morbidities into survivorship care. The reason is that worse physical and mental health have a negative impact on survival outcomes of breast cancer patients [16,17]. Therefore, identifying racial/ethnic differences and understanding survivors’ physical and mental health may assist providers in recommending the appropriate follow-up care with timely CRC screening.

Despite a few studies examining the importance of cancer screening uptake among breast cancer survivors with chronic diseases, prior research focuses primarily on mammography utilization for cancer recurrence [11,18]. Limited research examines the relationship between having chronic diseases and CRC screening utilization for secondary cancer prevention among breast cancer survivors. To date, only a single center study has observed that having hypertension was associated with 2.4-fold increased odds of having colonoscopy use among breast cancer survivors compared to those without hypertension [19]. To address this research gap, our study sought to examine the association between the presence of multi-morbidities and guideline-concordant CRC screening utilization. We also determined whether sociodemographic characteristics, quality of life, and cancer-related factors associated with screening uptake stratified by chronic diseases conditions among breast cancer survivors.

## 2. Materials and Methods

### 2.1. Study Design and Setting

We used data from the Behavioral Risk Factor Surveillance System (BRFSS). The BRFSS is a large cross-sectional survey administered annually by the Centers for Disease Control and Prevention (CDC) to about 400,000 adults, across all 50 states, the District of Columbia, Guam, and Puerto Rico. BRFSS utilizes a multistage cluster sampling technique to produce estimates representative of the US population. Data were collected via using survey questionnaire, including sociodemographic characteristics, cancer screening, cancer history, multiple chronic diseases, and health behaviors as well as health care access from noninstitutionalized adults aged ≥ 18 years residing in the US [20]. The respective health departments from each state grant Institutional Review Board (IRB) approval for the distribution and collection of data using the BRFSS and verbal consent as directed by the CDC survey. The BRFSS methods, sample selection, including the weighting procedure, are described elsewhere [20]. All BRFSS data and documentations are available at https://www.cdc.gov/brfss/index.html (accessed on 15 October 2022). Data extracted for this study were publicly available and de-identified, and thus considered exempt from IRB review.

### 2.2. Study Participants

**Figure A1:**
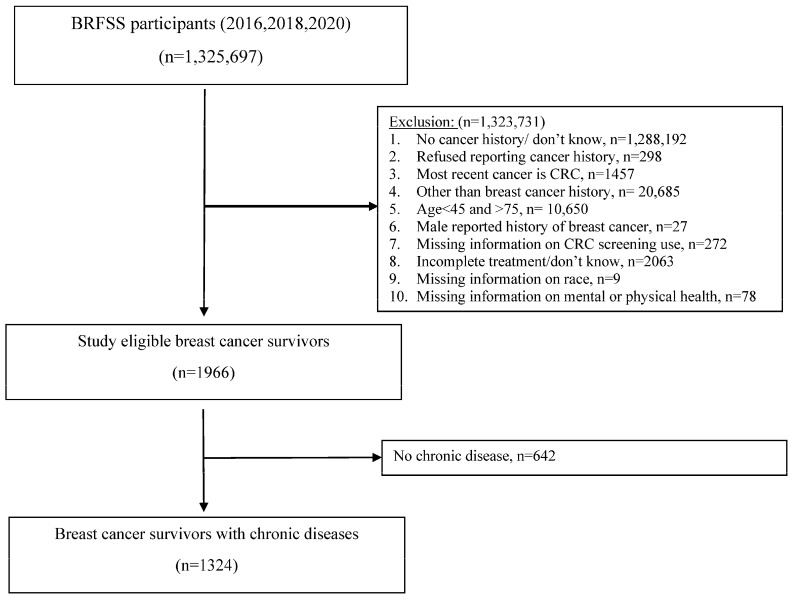
Selection process of eligible participants. Abbreviation: BRFSS, Behavioral Risk Factor Surveillance System; CRC, colorectal cancer.

A total of 1,325,697 respondents aged ≥ 18 years were included in the 2016, 2018, and 2020 BRFSS with available information on CRC screening utilization. To obtain an eligible study sample, we excluded respondents with no cancer history/don’t know (n = 1,288,192), refused reporting cancer history (n = 298), self-reported most recent cancer diagnosis of CRC (n = 1457), those with a history of cancer other than breast cancer (n = 20,685), male reported history of breast cancer (n = 27), missing information on CRC screening utilization (n = 272), and incomplete cancer treatment (n = 2063). Male breast cancer survivors were excluded from analysis due to low representative sample in breast cancer. According to the American Cancer Society (ACS) and the U.S. Preventive Services Task Force (USPSTF) CRC screening recommendations [21,22], adults less than 45 or greater than 75 years of age were excluded (n = 10,650). Finally, individuals with missing information on race/ethnicity (n = 9), self-reported mental or physical health (n = 78), and having no chronic disease (n = 642) were also excluded, corresponding to a total of 1324 breast cancer survivors with complete treatment and any chronic diseases as the final sample of the study (flowchart in Appendix A Figure A1).

### 2.3. Guideline-Concordant CRC Screening: Outcome of Interest

Receipt of guideline-concordant CRC screening was our primary outcome of interest. We determined whether breast cancer survivors had ever received a guideline-concordant CRC screening based on American Cancer Society (ACS) and the United States Preventive Service Task Force (USPSTF) recommendations [21,22]. We defined cancer survivors with a guideline-concordant CRC screening as those that (1) had a colonoscopy within 10 years, (2) sigmoidoscopy within 5 years, or (3) fecal occult blood test (FOBT) within a year. Survivors that received a colonoscopy more than 10 years, sigmoidoscopy more than 5 years, FOBT more than a year, or never used any of these three CRC screening options were categorized as having no guideline-concordant CRC screening.

### 2.4. Chronic Diseases Conditions: Primary Exposure of Interest

Chronic diseases were selected based on availability within the BRFSS study, including diabetes, coronary heart disease (CHD) or myocardial infarction (MI), stroke, chronic obstructive pulmonary disease (COPD), emphysema or chronic bronchitis, arthritis, depressive disorder, or kidney diseases (not including kidney stones, bladder infection, or incontinence) [23,24]. Further, we calculated the number of chronic diseases and categorized them into a three-level variable including (1) one, (2) two, and (3) three or more chronic diseases (also termed as multi-morbidities).

### 2.5. Covariates of Interest: Sociodemographic Characteristics, Quality of Life, and Cancer-Related Factors

Our primary covariates of interest were quality of life (mental and physical health) and race/ethnicity (non-Hispanic White, non-Hispanic Black, non-Hispanic, others/Hispanic). Mental and physical health were defined by using two BRFSS questions, which are “Now thinking about your mental health, which includes stress, depression, and problems with emotions, for how many days during the past 30 days was your mental health not good?” and “Now thinking about your physical health, which includes physical illness and injury, for how many days during the past 30 days was your physical health not good?”. These two questions with continuous answers (i.e., number of days) were then categorized as 0–13 days (termed as infrequent) and 14–30 days (termed as frequent) of poor health. Physicians and researchers commonly use this cut point as a marker for depression and anxiety disorders and unhealthy physical days [25,26].

The remaining non-primary covariates of interest were adjusted in multivariable models. For sociodemographic characteristics, we included age (45–59, 60–69, or 70–74), education (high school graduate or lower education attainment, some college graduate or higher), and income (less than $50,000 or $50,000 or more). For cancer-related factors, we included cancer treatment insurance (yes or no), current provider type (general practice or non-general practice), treatment summary (yes or no), and receipt of follow-up care plans (yes or no).

### 2.6. Statistical Analysis

Sampling weights provided in the 2016, 2018, and 2020 BRFSS data that adjust for unequal selection probabilities, survey non-response, and oversampling were used to account for the complex sampling design and to obtain population-based estimates. Weighted estimates and corresponding 95% confidence intervals (CI) were used to describe the characteristics of the study population. Cross-tabulation of frequency and weighted percentages were conducted to describe the association of the presence of chronic diseases, sociodemographic characteristics, quality of life, and cancer-related factors with guideline-concordant CRC screening utilization among breast cancer survivors by using weighted Rao–Scott chi-square tests. Weighted multivariable logistic regression model was applied to assess the association between guideline-concordant CRC screening utilization and level of chronic disease conditions, adjusted for all variables (sociodemographic characteristics, quality of life, and cancer-related factors). Further, we performed subpopulation analyses within each level of chronic disease conditions (1, 2, or 3+) to determine whether sociodemographic characteristics, quality of life, and cancer-related factors were associated with screening uptake stratified. Finally, we tested interaction between mental/physical health status and receipt of follow-up care plans. Such modification will enable the potential explanation for patient and physician communication regarding their quality of life resulted in adherence to CRC screening recommendation. Other non-primary covariates of interest with missing data, do not know, or refused responses are presented in Appendix A Table A1 and removed for all multivariable analyses. All results were reported as odds ratios (ORs) and the associated 95% confidence intervals (CIs). The level of statistical significance was set at an alpha level of 0.05 and the *p*-values were based on two-sided probability tests. We used SAS Version 9.4, SAS Institute Inc., Cary, NC, USA to perform all the analyses.

## 3. Results

### 3.1. CRC Screening Utilization

In Table 1, we present and describe the sociodemographic characteristics, quality of life, and cancer-related factors on guideline-concordant CRC screening stratified by level of chronic disease conditions. In general, CRC screening utilization was slightly higher among breast cancer survivors with multi-morbidities (88.3%) compared to those with one (84.4%) or two (85.4%) chronic diseases. Survivors with more chronic diseases demonstrated greater CRC screening use (*p*-value = 0.044). When exploring sociodemographic characteristics, greater CRC screening use was observed in non-Hispanic Black survivors regardless of chronic disease conditions (*p*-value < 0.001). Among cancer survivors with one chronic disease, higher CRC screening use was observed in those who had some college or higher degree (87.6%) and earned $50,000 or more annually (88.8%) (*p*-value < 0.05). In quality of life, lower screening use was observed in survivors with frequent poor mental health regardless of chronic disease conditions (*p*-value < 0.001) and survivors with frequent poor physical health when they had one chronic disease (67.1%; *p*-value < 0.001). Further, we observed that 93.1% and 92.6% of breast cancer survivors with multi-morbidities had CRC screening use when they received a treatment summary and a follow-up care plan (*p*-value < 0.001), respectively.

### 3.2. The Relationship of Multi-Morbidities on CRC Screening Utilization

Table 2 examines the association between the presence of chronic diseases and CRC screening use, adjusted for sociodemographic characteristics, quality of life, and cancer-related factors. In our full model, survivors with multi-morbidities (OR, 2.10; 95% CI, 1.11–3.98) exhibited greater odds of having CRC screening compared to breast cancer survivors with only one chronic disease. In sub-population analyses within three chronic disease conditions, we found that race/ethnicity was associated with CRC screening use (*p*-value < 0.001). Non-Hispanic Black survivors were more likely to have CRC screening use compared to non-Hispanic White survivors regardless of chronic disease conditions (one chronic disease: OR, 4.54; 95% CI, 1.08–19.11; two chronic diseases: OR, 5.17; 95% CI, 2.49–10.74; multi-morbidities: OR, 14.07; 95% CI, 5.61–35.27). However, non-Hispanic others/Hispanic survivors had lower odds of CRC screening use compared to non-Hispanic White when they had one disease (OR, 0.04; 95% CI, 0.01–0.12) or multi-morbidities (OR, 0.09; 95% CI, 0.05–0.17). Among survivors with multi-morbidities, those with frequent poor physical health were 3.32-times higher odds of receiving CRC screening (OR, 3.32; 95% CI, 1.57–7.00); conversely, those survivors with frequent poor mental health exhibited the lower odds of having CRC screening (OR, 0.33; 95% CI, 0.14–0.74).

In Table 3, we examine the interaction between mental/physical health and receipt of follow-up care plans among breast cancer survivors with multi-morbidities. Among those with frequent poor mental health, they were 92% less likely to be screened for CRC when they did not receive follow-up care plans (OR, 0.08; 95% CI, 0.03–0.21). In physical health status, survivors received follow-up care plans were 4.87-fold in infrequent group (OR, 4.87; 95% CI, 1.62–14.63) and 8.27-fold in frequent group (OR, 8.27; 95% CI, 2.90–23.56) more likely to have CRC screening utilization.

## 4. Discussion

Although receiving appropriate cancer screening for secondary CRC prevention is recommended due to increased risk among breast cancer survivors [12,19], research on CRC screening utilization among breast cancer survivors, particularly for those with multi-morbidities, is extremely lacking. Prior research examined the association between multi-morbidities and cancer screening among breast cancer survivors, predominantly focusing on mammography utilization [11,18]. To our best knowledge, this study is the first to examine guideline-concordant CRC screening use and determine key factors among breast cancer survivors with chronic disease conditions using a nationally representative sample of US participants.

### 4.1. Chronic Disease Conditions on CRC Screening

Overall, nearly half of survivors had at least two chronic diseases, including 27.1% and 19.3% of them with two diseases and multi-morbidities, respectively. Survivors with multi-morbidities were associated with greater CRC screening use (*p*-value < 0.05). There were 88.3% of survivors with multi-morbidities that had greater CRC screening use compared to those with one or two diseases. This was consistent with a prior 2020 BRFSS study [12]. Further, findings from Dash et al. reported that 46.9% of breast cancer survivors with hypertension had greater colonoscopy use compared to those without hypertension (30.1%). Comparable results in higher colonoscopy use from Dash et al. were also found in breast cancer survivors with diabetes compared to those without (48.4% vs. 38.8%) [19]. These together imply that breast cancer survivors with chronic diseases have potential to access timely cancer screening due to regular doctor visits for chronic disease management [27].

In multivariable analysis, we observed that breast cancer survivors with multi-morbidities were two-fold more likely to be screened for CRC adjusting for sociodemographic characteristics, quality of life, and cancer-related factors. Compared to the results from a 2020 BRFSS study, having chronic diseases was associated with greater CRC screening use than those without any chronic disease among breast cancer survivors [12]. Dash et al. also reported that breast cancer survivors with hypertension were 2.4-fold more likely to have timely colonoscopy [19]. An explanation from these findings is that the increased risk of chronic diseases (such as heart failure, heart disease, hypertension, or diabetes) is associated with oncological therapies, including the type of drug or radiotherapy/chemotherapy [28,29]. This might have the potential for increasing doctor visits leading to greater adherence to other preventive care, such as CRC screening. However, we were unable to examine the association with treatment modalities due to unavailable information from the BRFSS survey. Due to the shared risk factors (e.g., unhealthy diet or smoking) in chronic diseases and breast cancer [30,31], more research should further investigate what temporal patterns of chronic disease prevalence. This has implications for applying appropriate chronic disease management strategies in cancer survivorship care.

### 4.2. Other Covariates of Interest in CRC Screening

In sub-population analysis, we found that non-Hispanic Black breast cancer survivors were more likely to have screening use regardless of chronic disease conditions. Given that limited studies have examined the relationship between racial/ethnic differences in CRC screening behaviors among breast cancer survivors with chronic diseases, it was not possible to directly compare the study results. A prior study reported that Black breast cancer survivors were 1.3 times more likely to receive colonoscopy screening in breast cancer albeit they found no statistical significance [19]. This lack of association may be due to inadequate representation of the sample from a single center. When exploring findings from the US population, Shay et al. reported that Black cancer survivors were 1.2 times more likely to be guideline-concordant CRC screening in breast, cervical, colorectal, prostate, melanoma, and other cancers [13]. Among Hispanic survivors, Shay et al. found Hispanic survivors were 90% less likely to receive guideline-concordant CRC screening after complete treatment than White survivors [13]. Our finding also confirmed this association: other non-Hispanic/Hispanic survivors were 96% and 91% less likely to be screened for CRC when they had one chronic disease and multi-morbidities, respectively. Those findings suggest that racial/ethnic differences in CRC screening exist in cancer survivors. More research is required to understand how the influence of multi-morbidities on screening behaviors, particularly for women of color in breast cancer.

Another important insight from our findings is that survivors with frequent poor physical health and receipt of follow-up care plans were positively associated with CRC screening uptake. Because there is no study that particularly examined the influence of physical health and receipt of follow-up care plans among breast cancer survivors on CRC screening, it is difficult to compare the results directly. Yet, early evidence reported that survivors with one to two comorbidities were significantly associated with poor physical health in breast, prostate, and colorectal cancers within three years of an initial diagnosis [32]. Deshpande et al. also demonstrated that chronic disease burden was negatively associated with physical functioning in breast cancer [33]. The presence of multi-morbidities may lead to increased healthcare utilization [34] and thus having the possibility to receive follow-up instructions on chronic disease management and evaluation. Furthermore, improved adherence to CRC screening recommendations can be achieved through patient-centered communication that prioritizes continuity of care. Conversely, survivors with frequent poor mental health were 67% less likely to be screened for CRC when they had multi-morbidities in our analyses. It is possible that those survivors with poor mental health did not receive appropriate care [35]; consequently, unmet mental health needs may affect their decisions to access cancer screening [36].

Follow-up care plans have potential for patient-centered communication. We further examined the interaction between mental/physical health and receipt of follow-up care plans on screening uptake. Among survivors with frequent poor mental health, they were 98% less likely to be screened for CRC when they did not receive follow-up care plans. While there was evidence that breast cancer survivors commonly experience unmet needs in psychological well-being, more research is required in evaluating best practice models of care that address their concerns [35,37,38]. Further, we found that survivors with follow-up care plans were more likely to receive CRC screening regardless of physical health status. It is possible that those breast cancer survivors had a primary care provider involved in their follow-up care, which has the potential for receiving more appropriate care (such as CRC screening) in terms of preventive services [39]. Thus, those results signify the importance of chronic disease management considering the mental and physical health status of breast cancer survivors. Approach, such as patient navigation programs, may be a potential intervention that can increase access to appropriate follow-up and other preventive care [36]. Such programs will further assist in improving linkages across health care and social service providers to address timely CRC screening and specific needs (such as mental/physical health) simultaneously by using coordinated care models.

Our findings from a nationally representative sample may contribute to the limited number of studies evaluating the relationship between the presence of multi-morbidities and secondary cancer prevention in breast cancer. Despite this study’s strengths, a few limitations should be noted. First, the survey question designed for cancer survivorship was to collect respondents’ most recent cancer type. Therefore, if participants had multiple cancers and the most recent one was not CRC, we could not exclude them from this study. CRC screening utilization may be overestimated for those with CRC history due to frequent follow-up CRC screening to detect recurrence. Second, we applied a cross-sectional analysis; therefore, we are unable to assess temporal relationship (i.e., did chronic diseases occur before or after breast cancer diagnosis). Third, chronic disease conditions were self-reported in our study, and this could potentially lead to underreporting of medical conditions. Finally, we conducted analyses on breast cancer survivors across a wide range of follow-up times since breast cancer diagnosis. We did this because the BRFSS survey does not serve as a cancer surveillance database and does not collect information regarding cancer prognosis and progression. The influence of multiple chronic diseases on screening decisions may be different across various phases of survivorship (e.g., short-term, or long-term).

## 5. Conclusions

Overall, having multi-comorbidities was positively associated with guideline-concordant CRC screening use among breast cancer survivors. Other factors associated with screening uptake include race/ethnicity, mental and physical health, and receipt of follow-up care plans across different chronic diseases conditions. Findings from this study suggest the importance of chronic disease management considering mental/physical health status and other factors (race/ethnicity or receipt of follow-up care plans) in cancer survivorship care. Utilization of patient navigation programs may also increase adherence to CRC screening recommendations and other follow-up care simultaneously among breast cancer survivors.

## Figures and Tables

**Table 1:** Descriptive statistics of sociodemographic characteristics, quality of life, cancer related factors, and CRC screening utilization (outcome of interest) by chronic disease conditions.

	Total (n = 1324)	One (n = 702)		Two (n = 388)		Three or More (n = 234)	
**Having CRC screening ^a^**		84.4%		85.4%		88.3%	
		CRC screening ^a^ n (%) ^b^	*p*-value ^e^	CRC screening ^a^ n (%) ^b^	*p*-value ^e^	CRC screening ^a^ n (%) ^b^	*p*-value ^e^
Sociodemographic characteristics							
Age			0.069		0.118		0.776
45–59	277 (27.7%)	120 (84.1%)		53 (85.8%)		43 (87.8%)	
60–69	638 (46.1%)	291 (81.4%)		170 (82.3%)		83 (89.7%)	
70–74	409 (26.1%)	179 (90.0%)		107 (91.8%)		67 (86.9%)	
Race/Ethnicity			<0.001		<0.001		<0.001
Non-Hispanic White	1070 (77.5%)	500 (87.7%)		264 (83.1%)		143 (87.5%)	
Non-Hispanic Black	160 (15.4%)	62 (94.6%)		50 (95.8%)		31 (97.1%)	
Non-Hispanic Others/Hispanic ^c^	94 (7.0%)	28 (33.4%)		16 (84.9%)		19 (72.7%)	
Education			0.009		0.700		0.106
High school or lower	387 (38.8%)	144 (78.4%)		158 (84.4%)		75 (86.0%)	
Some college or higher	935 (60.9%)	446 (87.6%)		121 (89.9%)		117 (91.1%)	
Income			0.003		0.202		0.686
Less than $50,000	583 (45.1%)	201 (79.4%)		359 (81.4%)		119 (88.1%)	
$50,000 or more	527 (40.1%)	292 (88.8%)		413 (89.1%)		46 (89.6%)	
Quality of life							
Mental health			<0.001		<0.001		<0.001
0–13 days	1157 (83.6%)	556 (87.8%)		288 (89.5%)		142 (92.2%)	
14–30 days	167 (16.4%)	34 (59.2%)		42 (60.5%)		51 (80.1%)	
Physical health			<0.001		0.570		0.695
0–13 days	1050 (78.8%)	529 (86.8%)		265 (86.1%)		103 (88.9%)	
14–30 days	274 (21.2%)	61 (67.1%)		65 (83.3%)		90 (87.6%)	
Cancer related factors							
Cancer treatment insurance			0.121		0.562		0.569
No	81 (6.5%)	27 (72.2%)		17 (81.8%)		16 (91.7%)	
Yes	1243 (93.5%)	563 (85.2%)		313 (85.7%)		177 (88.0%)	
Current provider type			0.859		0.530		0.599
Non-general practices ^d^	390 (30.5%)	171 (84.1%)		93 (86.4%)		59 (86.7%)	
General practices	907 (66.8%)	411 (84.8%)		235 (88.9%)		129 (88.8%)	
Treatment summary			0.427		0.469		<0.001
No	482 (36.6%)	192 (82.3%)		121 (83.7%)		85 (82.6%)	
Yes	735 (55.8%)	351 (85.0%)		186 (86.5%)		90 (93.1%)	
Follow-up care plan			0.544		0.517		<0.001
No	195 (13.4%)	84 (80.9%)		46 (83.7%)		26 (60.0%)	
Yes	1099 (84.1%)	493 (84.7%)		280 (87.4%)		160 (92.6%)	

Abbreviation: CRC, colorectal cancer. ^a^ Data on those without CRC screening within three chronic disease conditions use are not shown in the table. ^b^ Data shown as frequency and weighted percentages. There are missing values for education, income, provider, treatment summary, and follow-up care plan (Appendix A Table A1). ^c^ Other non-Hispanic include Asian, American Indian/Alaskan Native, and others. ^d^ Non-general practices include surgeon, oncology, urologists, and others. ^e^ Weighted chi-square test was used. All weighted percentages are based on row total.

**Table 2:** The association between sociodemographic characteristics, cancer related factors, quality of life, and CRC screening use and stratified by chronic disease conditions.

	Total	One	Two	Three or More
	OR (95% CI) ^a^	OR (95% CI) ^a^	OR (95% CI) ^a^	OR (95% CI) ^a^
Chronic disease conditions				
One	Reference	NA	NA	NA
Two	1.36 (0.74, 2.50)	NA	NA	NA
Three or more	*2.10* (*1.11*, *3.98*)	NA	NA	NA
Sociodemographic characteristics				
Age				
45–59	Reference	Reference	Reference	Reference
60–69	1.30 (0.74, 2.28)	1.35 (0.66, 2.76)	1.12 (0.42, 3.01)	2.27 (0.84, 6.11)
70–74	1.48 (0.75, 2.91)	1.70 (0.65, 4.45)	1.81 (0.68, 4.82)	0.84 (0.36, 1.92)
Race/Ethnicity				
Non-Hispanic White	Reference	Reference	Reference	Reference
Non-Hispanic Black	*4.72* (*1.69*, *13.19*)	*4.54* (*1.08*, *19.11*)	*5.17* (*2.49*, *10.74*)	*14.07* (*5.61*, *35.27*)
Non-Hispanic Others/Hispanic	*0.15* (*0.08*, *0.27*)	*0.04* (*0.01*, *0.12*)	1.92 (0.55, 6.75)	*0.09* (*0.05*, *0.17*)
Education				
High school or lower	Reference	Reference	Reference	Reference
Some college or higher	1.31 (0.84, 2.06)	0.75 (0.42, 1.33)	1.49 (0.80, 2.78)	*4.79* (*2.19*, *10.47*)
Income				
Less than $50,000	Reference	Reference	Reference	Reference
$50,000 or more	*1.50* (*0.87*, *2.61*)	*1.98* (*1.08*, *3.63*)	1.48 (0.71, 3.08)	0.99 (0.33, 2.95)
Quality of life				
Mental health				
0–13 days	Reference	Reference	Reference	Reference
14–30 days	0.44 (0.25, 0.76)	0.68 (0.35, 1.31)	0.82 (0.29, 2.37)	*0.33* (*0.14*, *0.74*)
Physical health				
0–13 days	Reference	Reference	Reference	Reference
14–30 days	1.05 (0.59, 1.88)	1.07 (0.51, 2.23)	0.66 (0.29, 1.51)	*3.32* (*1.57*, *7.00*)
Cancer related factors				
Cancer treatment insurance				
No	Reference	Reference	Reference	Reference
Yes	*1.03* (*0.52*, *2.02*)	*3.30* (*1.38*, *7.89*)	0.51 (0.22, 1.17)	*0.02* (*0.002*, *0.32*)
Current provider type				
Non- general practices	Reference	Reference	Reference	Reference
General practices	1.06 (0.60, 1.86)	0.75 (0.34, 1.62)	1.81 (0.66, 4.94)	2.16 (0.86, 5.45)
Treatment summary				
No	Reference	Reference	Reference	Reference
Yes	0.94 (0.61, 1.45)	0.92 (0.51, 1.68)	1.25 (0.57, 2.78)	*3.20* (*1.29*, *7.92*)
Follow-up care plan				
No	Reference	Reference	Reference	Reference
Yes	*3.05* (*1.68*, *5.53*)	*2.23* (*1.07*, *4.66*)	2.07 (0.84, 5.07)	*4.89* (*1.92*, *12.47*)

Abbreviation: NA, non-applicable; CRC, colorectal cancer; OR, odd ratio. Italicized text indicates statistically significant result. ^a^ Weighted logistic regression was used in all models.

**Table 3:** Modification of the effect of receiving a follow-care plan on CRC screening utilization by mental and physical health status among breast cancer survivors with multi-morbidities ^a^.

	Follow-Up Care Plan Yes		Follow-Up Care Plan No		
	n (%) ^b^ with/without CRC screening	OR (95%CI)	n (%) ^b^ with/without CRC screening	OR (95%CI)	*p*-value for receiving follow-up care plans within strata of mental/physical health
**Mental health**					
0–13 days	828 (85.4%)/140 (83.4%)	1.21 (0.55, 2.68)	137 (12.5%)/27 (15.2%)	Reference	0.625
14–30 days	105 (87.0%)/26 (60.9%)	1.44 (0.64, 3.22)	19 (9.9%)/12 (29.8%)	*0.08* (*0.03*, *0.21*)	<0.001
**Physical health**					
0–13 days	758 (85.2%)/122 (72.7%)	*4.87* (*1.62*, *14.63*)	120 (12.7%)/26 (21.5%)	Reference	0.006
14–30 days	175 (87.2%)/44 (81.1%)	*8.27* (*2.90*, *23.56*)	36 (10.0%)/13 (18.5%)	2.61 (0.88,7.75)	0.028

Abbreviation: CRC, colorectal cancer; OR, odd ratio. Italicized text indicates statistically significant result. ^a^ ORs was adjusted for sociodemographic characteristics, quality of life, and cancer related factors. Weighted logistic regressions were used. ^b^ Data shown as frequency and weighted percentages. CRC screening rates from missing values of follow-up care plan are not presented.

## Data Availability

The datasets generated during the current study are available in the Center for Disease Control, and Prevention repository, https://www.cdc.gov/brfss/ (accessed on 15 October 2022).

## Appendix A

**Table A1:** Missing values for each factor among breast cancer survivors ^a^.

	Chronic Diseases One to Two (n = 702)	Chronic Diseases One to Two (n = 388)	Chronic Diseases Three or More (n = 234)
	n	n	n
Education	0	1	1
Income	118	60	36
Provider	15	5	7
Treatment summary	56	27	24
Follow-up care plan	17	5	8

^a^ Chronic disease conditions, age, race/ethnicity, mental and physical health, and cancer treatment coverage do not have missing values.
